# Muscle index, weight loss, and tumor type predict adjuvant therapy failure in periampullary cancer

**DOI:** 10.3389/fnut.2026.1868235

**Published:** 2026-07-27

**Authors:** Zhe Liu, Shi-Wei Yang, You-Wei Ma, Li-Fang Si, Fei Yang, Jun-Xi Zhang, Qin-Xin Li, MiRiBai Tuo, Si-Wei Guo, Wen-li Xu, Xian-Liang Li, Qiang He, Dong-Dong Han, Ji-Qiao Zhu

**Affiliations:** 1 Department of Hepatobiliary and Pancreaticosplenic Surgery, Beijing Chaoyang Hospital, Capital Medical University, Beijing, China; 2 Department of Hepatobiliary Surgery, China-Japan Friendship Hospital, Beijing, China; 3 Department of Radiology, Beijing Chaoyang Hospital, Capital Medical University, Beijing, China

**Keywords:** cachexia, disease-free survival, mediation analysis, nutritional risk, pancreaticoduodenectomy, sarcopenia

## Abstract

**Background:**

Periampullary cancers frequently induce malnutrition and sarcopenia, which impair treatment tolerance and survival. Current nutritional assessment tools evaluate host status in isolation, ignoring the modifying effect of tumor biology. We developed and validated a simple nutritional risk score integrating host and tumor domains to identify high-risk patients and quantify the extent to which the association between preoperative risk and poor outcomes is mediated through a sequential pathway.

**Methods:**

This dual-center retrospective cohort study included 533 patients who underwent pancreaticoduodenectomy. Using a time-ordered split, patients were assigned to training (*n* = 261), internal validation (*n* = 112), and temporal validation (*n* = 160) sets. Least Absolute Shrinkage and Selection Operator (LASSO)-Cox regression selected predictors for disease-free survival (DFS). The final model was validated internally and temporally, with head-to-head comparisons against 12 established nutritional scores. Formal sequential mediation analysis quantified the pathway from preoperative nutritional risk to postoperative complications, then to adjuvant therapy delay, and ultimately to reduced DFS.

**Results:**

The cohort had mean age 64.0 ± 9.8 years, 57.8% male. Median follow-up was 23 months. During follow-up, 261 recurrence or death events (49.0%) occurred; 1-year mortality was 55.7% (95%CI 51.5-59.9%). LASSO-Cox identified three independent predictors: total muscle index (TMI, mm^2^/m^2^), percent weight loss (>5% vs. ≤ 5%), and tumor type. The model showed good discrimination: C-index 0.783 (training), 0.718 (internal validation), and 0.702 (temporal validation). It significantly outperformed all 12 nutritional scores (all *P* < 0.001). High-risk patients had worse DFS (log-rank *P* < 0.0001) and a 3.29-fold higher risk of major complications (95% CI: 1.23–9.46). Complications led to a 22.27-fold higher risk of adjuvant therapy delay (95% CI: 6.70–90.28), which in turn reduced DFS (HR 2.66, 95% CI: 1.39–5.09). Mediation analysis showed that 20.9% (95% CI: 5.1–36.7%) of the total effect of high nutritional risk on DFS was mediated through this complication-to-delay pathway.

**Conclusions:**

A simple three-variable score (muscle index, weight loss >5%, tumor type) enables preoperative identification of high-risk periampullary cancer patients. One-fifth of the excess recurrence risk may be mediated through a modifiable cascade of complications leading to adjuvant therapy delays, providing a clear rationale for targeted nutritional prehabilitation and perioperative interventions.

## Introduction

Pancreaticoduodenectomy (PD) offers the sole curative potential for pancreatic head and periampullary malignancies (1), yet outcomes remain starkly heterogeneous with 5-year survival ranging from < 5% to 45% (2–5). This profound variability cannot be fully explained by conventional tumor-centric factors, but a more immediate and potentially modifiable failure occurs in the weeks after surgery: up to 40% of patients who undergo successful resection never receive the adjuvant therapy that guidelines recommend. The host's physiological state is a critical determinant of prognosis, a dimension that current assessment paradigms fail to adequately capture (6, 7).

A patient's physiological reserve, particularly their nutritional status and body composition, has emerged as a critical determinant of resilience (8). The anatomical predisposition of these tumors to cause biliary obstruction and malabsorption leads to a high prevalence of malnutrition and sarcopenia (9). These conditions are not merely markers of advanced disease but are active contributors to a catabolic state that can impair immune competence, amplify postoperative morbidity, and critically, erode the physiological buffer required to tolerate and complete adjuvant therapy (10).

Numerous nutritional assessment tools have been employed to capture this vulnerability, yet the literature reveals striking inconsistency. While some studies report that scores such as the Nutritional Risk Screening Score 2002 (NRS2002) (11) and the prognostic nutritional index (PNI) (12) effectively stratify prognosis after PD, others, including head-to-head comparisons of twelve tools, found that either only one (the Subjective Global Assessment, SGA) (13) or none (14) were predictive of survival. This discordance reflects a conceptual flaw: these tools treat host factors in isolation while neglecting the modifying effect of tumor biology. For instance, the prognostic impact of weight loss likely differs between indolent ampullary carcinoma (AC) and aggressive pancreatic ductal adenocarcinoma (PDAC) (15–17), a distinction that existing nutritional tools systematically ignore.

Recent advances in understanding the ‘tumor macroenvironment' support integrating host and tumor domains, positing that systemic host physiology and tumor biology are engaged in constant bidirectional crosstalk (18). We hypothesized that objective measures of host physiological reserve—total muscle index (TMI) as a structural biomarker and percent weight loss as a dynamic indicator of metabolic depletion—must be interpreted through the lens of tumor biology (type) to stratify risk for postoperative complications and subsequent adjuvant therapy disruption.

Therefore, this study aimed to: (1) develop an integrative risk score combining tumor pathology with host physiology; (2) validate it through temporal validation; (3) conduct head-to-head comparisons against conventional nutritional tools; and (4) use formal mediation analysis to quantify the extent to which postoperative complications and adjuvant therapy disruption sequentially mediate the survival disadvantage of high-risk patients.

## Materials and methods

### Study population and design

While pancreatic head and periampullary carcinomas exhibit distinct biological behaviors, they share a common surgical approach (PD), anatomical location, and pathophysiology leading to nutritional depletion (e.g., biliary obstruction, malabsorption). This study aimed to develop a prognostic tool applicable to the shared surgical population undergoing PD. Our core hypothesis is that interpreting objective host physiology through the lens of tumor biology creates a superior, generalizable predictive signature. As later validated in the results, the final model demonstrated robust discriminative ability within each major tumor subtype, and the risk scores it generated perfectly aligned with the established gradient of tumor aggressiveness (PDAC > CDP/DBC > AC), confirming its biological coherence and clinical utility across this spectrum of diseases.

This dual-center, retrospective cohort study consecutively enrolled patients with pancreatic head and periampullary carcinoma who underwent PD at Beijing Chaoyang Hospital from January 2013 to July 2024, and at China-Japan Friendship Hospital from January 2019 to July 2024. All PD procedures were performed by a consistent team of experienced pancreatic surgeons using standardized techniques for resection and reconstruction, following established perioperative management protocols (19–22). All surgeons had performed >30 PD procedures annually.

### Variable definitions

Percentage weight loss was calculated using the formula: [(Pre-illness weight - Preoperative weight) / Pre-illness weight] x 100%. Pre-illness weight was defined as the patient's self-reported stable body weight approximately 6 months before surgery or before the onset of cancer-related symptoms. To mitigate recall bias, this information was cross-referenced and supplemented by: (1) documented weights in prior medical records (when available within 6–12 months before symptom onset), and (2) clinical interview by a dietitian to identify and clarify implausible self-reports (e.g., extreme values inconsistent with clinical presentation).

Postoperative abdominal complications were documented and classified according to internationally accepted criteria, including the International Study Group of Pancreatic Surgery (ISGPS) definitions for postoperative pancreatic fistula (POPF), delayed gastric emptying (DGE), and hemorrhage (23–25), as well as the International Study Group of Liver Surgery (ISGLS) criteria for bile leak (26).

Adjuvant chemotherapy delay was consistently defined as either: (1) failure to initiate any adjuvant chemotherapy within 90 days postoperatively, or (2) complete omission of indicated adjuvant chemotherapy. ‘Indicated' adjuvant therapy was rigorously defined *post-hoc* for all patients based on a consensus review of histopathology and staging data, applying contemporary Chinese and international guidelines for periampullary cancers prevalent during the patient's treatment era. This determination was made independently of the occurrence of complications or nutritional status by a study panel comprising a surgical oncologist, a medical oncologist, and a pathologist.

## Results

### Patient cohort and characteristics

**Figure 1:**
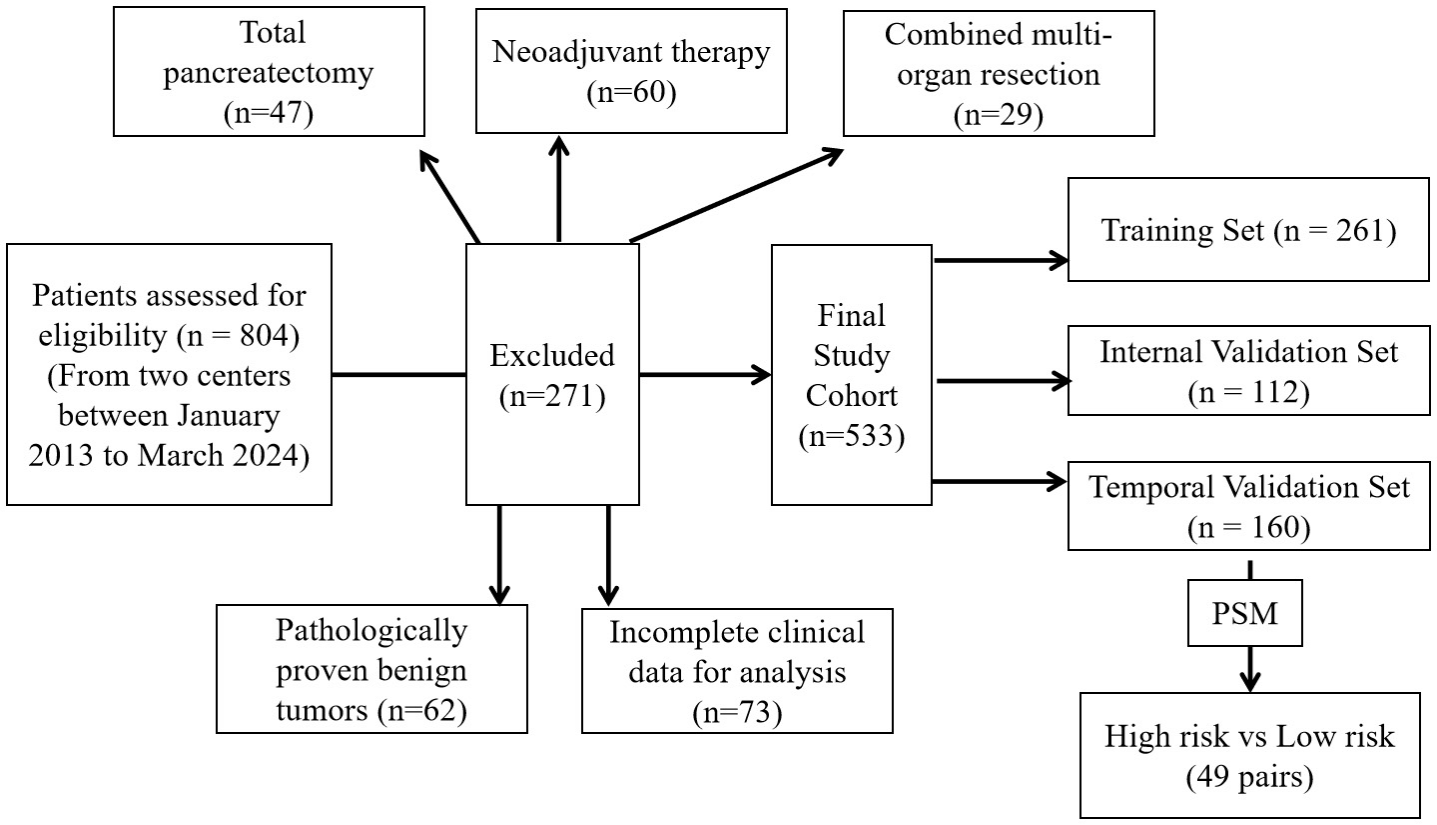
Study cohort selection flow diagram. The cohort was partitioned using a time-ordered split strategy, with the most recent 30% of patients constituting the temporal validation set.

Given the shared surgical approach and perioperative management for pancreatic head and periampullary malignancies, we analyzed these histologic subtypes collectively. The study cohort comprised 533 patients who met the inclusion criteria, with detailed patient flow illustrated in the Consolidated Standards of Reporting Trials (CONSORT) diagram (Figure 1). The cohort was partitioned into training (*n* = 261, 49%), internal validation (*n* = 112, 21%), and temporal validation (*n* = 160, 30%) sets using a time-ordered split strategy. Baseline characteristics are summarized in eTable 1 in the Supplement. The cohort had a mean age of approximately 64 years with balanced gender distribution (57.8% male). Tumor types included PDAC (39.4%), distal bile duct carcinoma (DBC, 35.6%), AC (16.3%), and carcinoma of the duodenal papilla (CDP, 8.6%). Most patients presented with stage II disease (54.2%), and R0 resection was achieved in 94.7% of cases. The median follow-up duration was 23 months (range: 6–134 months), and the 1-year mortality rate was 55.7% (95% CI: 51.5%−59.9%).

### Prediction model development and nomogram construction

**Figure 2:**
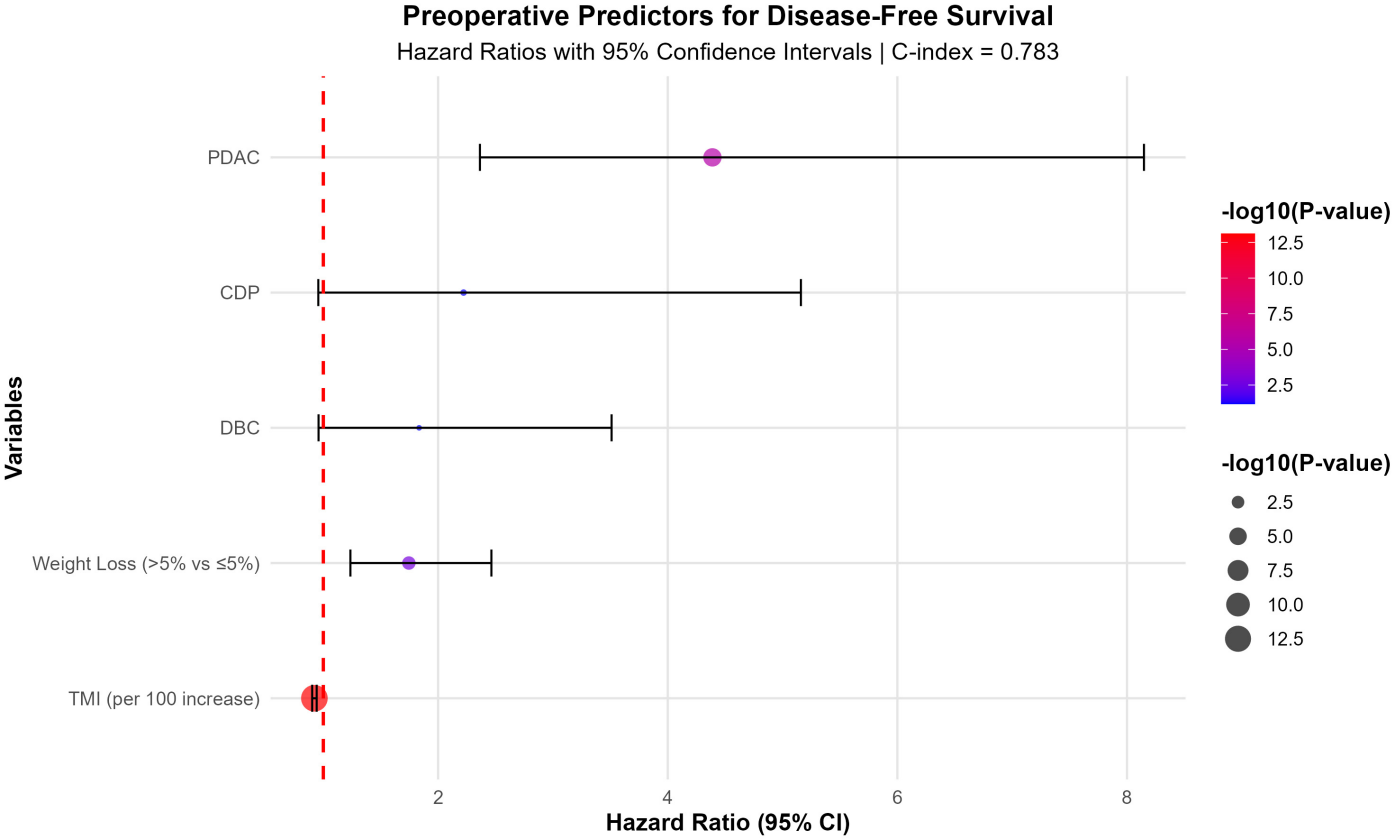
Forest plot of multivariable cox regression analysis. Hazard ratios with 95% confidence intervals for the three predictors in the final model. TMI (total muscle index) is presented per 100-unit increase, percent weight loss is dichotomized at the 5% threshold, and tumor types are compared to ampullary carcinoma (reference category). Point sizes and colors represent the -log10 (*P*-value), with larger and redder points indicating greater statistical significance. PDAC, pancreatic ductal adenocarcinoma; DBC, distal bile duct carcinoma; CDP, carcinoma of the duodenal papilla; TMI, total muscle index.

**Figure 3:**
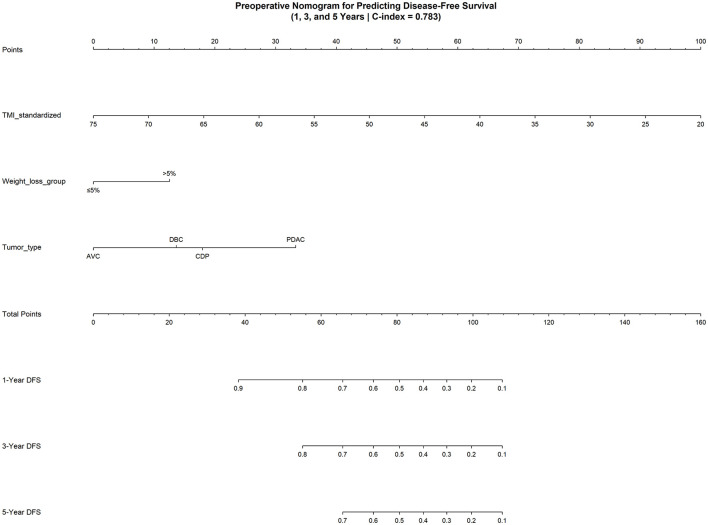
Nomogram for predicting disease-free survival. The nomogram integrates three predictors to estimate individual probabilities of 1-, 3-, and 5-year disease-free survival. To use: (1) Locate patient values on each variable axis, (2) Draw vertical lines to the points axis to determine score for each variable, (3) Sum all points, (4) Locate total points on the total points axis, and (5) Draw vertical line to read predicted survival probabilities at bottom scales. TMI, total muscle index; DFS, disease-free survival.

The LASSO-Cox regression analysis identified three independent predictors for DFS: TMI, percent weight loss (>5% vs. ≤ 5%), and tumor type. The variable selection process evaluated 22 candidate variables spanning demographic, clinical, laboratory, tumor-related, and body composition domains; the complete list of evaluated variables is provided in eTable 2. The comprehensive multivariable Cox regression analysis results are summarized in Figure 2. All selected variables exhibited significant associations with DFS. Higher TMI (per 100-unit increase) was associated with reduced recurrence risk (HR 0.922, 95% CI: 0.903–0.942, *P* < 0.001), while weight loss >5% significantly increased recurrence risk (HR 1.745, 95% CI: 1.236–2.464, *P* = 0.002). Regarding tumor type, using AC as the reference category, PDAC was associated with a substantially increased recurrence risk (HR = 4.389, 95% CI: 2.364–8.148, *P* < 0.001). Both DBC (HR = 1.835, 95% CI: 0.959–3.511, *P* = 0.067) and CDP (HR = 2.222, 95% CI: 0.957–5.160, P = 0.063) showed trends toward increased risk, though these associations did not reach statistical significance. We constructed an individualized prediction nomogram integrating these three predictors (Figure 3). This clinically practical tool enables estimation of 1-, 3-, and 5-year DFS probabilities, providing immediate risk stratification upon availability of routine clinical data.

### Internal and temporal validation of model performance

The predictive model underwent rigorous validation across multiple datasets. Discriminative performance across cohorts is detailed in eTable 3 in the Supplement. In brief, the model demonstrated strong discriminative capacity with a C-index of 0.783 (95% CI: 0.749–0.817). The internal validation set (*n* = 112, events = 66) maintained excellent performance with a C-index of 0.718 (95% CI: 0.665–0.771), indicating minimal overfitting. Most importantly, the temporal validation cohort (*n* = 160, events = 55) confirmed the model's generalizability with a C-index of 0.703 (95% CI: 0.620–0.786).

### Incremental value and comparative performance analysis

The three-parameter perioperative prediction model demonstrated superior performance compared to both baseline clinical models and existing nutritional assessment tools. Compared to a baseline model containing only tumor type (C-index = 0.597, 95% CI: 0.526–0.667), our comprehensive model showed significant improvement in discrimination (ΔC-index = 0.106, 95% CI: 0.055–0.157, *P* = 0.012), corresponding to a 17.8% relative increase in predictive accuracy (eFigure 5 in the Supplement). Reclassification analyses further confirmed model superiority (eFigure 6 in the Supplement), with a net reclassification improvement of 0.826 (*P* = 0.002) and integrated discrimination improvement of 0.150 (*P* = 0.001). The likelihood ratio test validated the overall model enhancement (χ^2^ = 41.01, *P* < 0.001), demonstrating consistent statistical significance across multiple validation metrics. To further elucidate the relative contributions of host factors versus tumor biology to model performance, we performed a nested model comparison in the temporal validation cohort (*n* = 160). The model containing only host factors (TMI and percent weight loss) achieved a C-index of 0.748 (95% CI: 0.689–0.807). In comparison, the model containing tumor type alone showed a C-index of 0.597 (95% CI: 0.526–0.667), and the full integrative model demonstrated a C-index of 0.776 (95% CI: 0.720–0.831). Notably, the host-only model substantially outperformed the tumor-only model (ΔC-index = 0.151), and the full model provided additional improvement over the host-only model (ΔC-index = 0.028). These findings confirm that host factors contribute the majority of the model's predictive power, while tumor type provides meaningful incremental value, supporting our conceptual framework of tumor-host integration rather than tumor type dominance. In head-to-head comparisons with conventional nutritional assessment tools (eFigure 7 in the Supplement), our model demonstrated unequivocal superiority, achieving a C-index of 0.702 that substantially exceeded all comparator scores.

### Risk stratification and association with postoperative outcomes

#### Robust risk stratification and survival disparities

**Figure 4:**
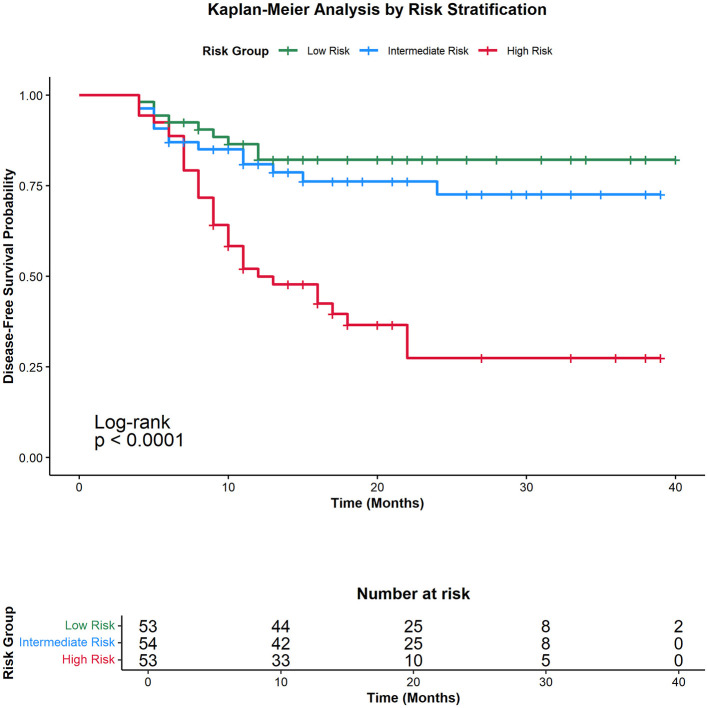
Disease-free survival stratification by nutritional risk groups in the temporal validation cohort. Kaplan-Meier curves demonstrate significant survival disparities among low- (*n* = 53), intermediate- (*n* = 54), and high-risk (*n* = 53) groups stratified by the our integrative model (log-rank *P* < 0.001).

Using the risk scores derived from the integrative model, patients in the temporal validation cohort (*n* = 160) were stratified into low- (*n* = 53), intermediate- (*n* = 54), and high-risk (*n* = 53) groups based on score tertiles. Kaplan-Meier analysis revealed significantly divergent DFS curves among these strata (Log-rank *P* < 0.0001; Figure 4).

#### Propensity-score matched validation and association with complications

**Figure 5:**
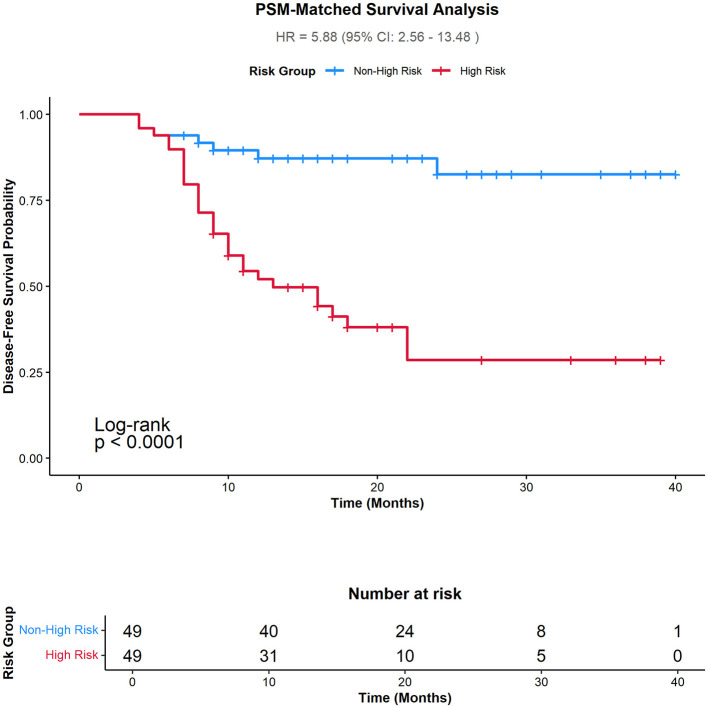
Propensity score-matched survival analysis confirming independent prognostic value. Kaplan-Meier curves after 1:1 matching (*n* = 98, 49 per group). Despite balanced baseline characteristics, high-risk patients maintained significantly worse disease-free survival (*P* < 0.001). PSM, propensity score matching.

To rigorously confirm that this stratification was independent of baseline confounders, we performed PSM in the temporal validation set. Details of the matching procedure and post-matching balance are provided in the Supplementary Methods and eTable 4, respectively. In the well-balanced PSM cohort, high-risk patients maintained a substantially increased hazard for recurrence (HR = 5.88, 95% CI: 2.56–13.48, *P* < 0.001; Figure 5) and exhibited a markedly higher rate of major postoperative complications (42.9% vs. 18.4%; OR = 3.29, 95% CI: 1.23–9.46, *P* = 0.015).

### Mediation analysis: elucidating the complication-to-treatment delay pathway

Formal sequential mediation analysis was performed to explore the mechanistic pathway. High nutritional risk was strongly associated with increased postoperative complications (OR = 3.29, 95% CI: 1.23–9.46, *P* = 0.015). Patients experiencing complications faced dramatically elevated risks of adjuvant therapy delays (OR = 22.27, 95% CI: 6.7–90.28, *P* < 0.001). While the wide CI: indicates heterogeneity in the magnitude of effect, it unequivocally supports a strong association between complications and therapy delays. This delay, in turn, significantly predicted worse DFS (HR = 2.66, 95% CI: 1.39–5.09, *P* = 0.003). Crucially, 20.9% of the total effect of nutritional risk on survival was mediated through this sequential pathway.

## Discussion

### Summary of key findings

This study demonstrates that an integrative risk score, available on postoperative day 7, identifies a patient phenotype with a 22-fold risk of adjuvant therapy failure after pancreaticoduodenectomy. Critically, formal mediation analysis reveals that 20.9% (95% CI: 5.1–36.7%) of the survival disadvantage in high-risk patients is sequentially mediated through a modifiable cascade: major postoperative complications leading to adjuvant therapy delay. Contextualizing objective host physiology within specific tumor biology provides superior prognostic stratification compared to conventional nutritional tools that assess host factors in isolation.

### Interpretation of the three risk components

The clinical relevance of the three score components is immediately clear to the multidisciplinary team. TMI, measurable from preoperative CT performed routinely for staging, serves as a structural biomarker of systemic protein-energy reserves, the physiological buffer against catabolic stress imposed by both surgery and chemotherapy (27, 28). Weight loss >5% captures dynamic metabolic depletion, the clinical hallmark of cancer-associated cachexia (29). Crucially, tumor type—available on postoperative day 7 - is not merely another prognostic factor but an effect modifier. Low muscle mass with weight loss in PDAC likely signifies tumor-driven metabolic catastrophe, with limited capacity for rapid reversal; the same findings in AC may reflect obstructive malnutrition with greater recovery potential after biliary decompression (15–17). Our model formalizes this biological distinction into a clinically actionable score, available at the exact moment when adjuvant therapy decisions are pending. A sensitivity analysis comparing nested models further confirmed that host factors (TMI and weight loss) contributed the majority of the model's predictive power (C-index = 0.748), with tumor type providing meaningful incremental value (ΔC-index = 0.028). This refutes the notion that tumor type simply dominates the model; rather, it serves as the biological context that modifies the prognostic impact of host physiology.

### Mediation analysis and clinical implications

The most important contribution of this study is moving beyond conventional prognostication to quantify a plausible and clinically actionable pathway. Formal mediation analysis demonstrates that 20.9% of the total survival disadvantage in high-risk patients is sequentially mediated through major complications leading to adjuvant therapy delay. This finding transforms the score from a passive prognosticator into a roadmap for clinical practice improvement. It identifies specific, modifiable junctures in perioperative care—anastomotic failure, infected collections, delayed recovery—that are not merely statistical correlates but potentially modifiable factors of downstream oncologic failure (30). By quantifying the proportion of the survival gap attributable to this pathway, we provide both the target and the expected effect size for quality improvement initiatives: intervening effectively on the complication-to-delay cascade could potentially close one-fifth of the survival disparity.

### Comparison with existing nutritional assessment tools

Our findings align with recent conceptual advances regarding the ‘tumor macroenvironment' (18) and extend previous work on sarcopenia (9, 10) and nutritional status (11–14) in pancreatic surgery by quantifying, for the first time, the mediating role of complications. Notably, our model outperformed all twelve conventional nutritional tools, including SGA, the sole tool previously identified as predictive in prospective head-to-head comparisons (13), reinforcing our central thesis that assessments ignoring tumor-host interaction are inherently incomplete.

### Translational perspective and implementation

From a translational perspective, this score was designed with implementation in mind. Available on postoperative day 7, it requires no additional tests or cost—only three data points already in the medical record: tumor histology, preoperative CT, and preoperative weight. This low implementation barrier was an explicit design goal. The score empowers multidisciplinary teams to risk-stratify at a pivotal decision node and deploy differential resource allocation: intensified post-discharge monitoring, aggressive nutritional support, early infectious surveillance, and proactive oncology navigation to safeguard adjuvant therapy timelines. The logical next step is a pragmatic effectiveness trial using the score to allocate enhanced recovery interventions specifically to high-risk patients, with co-primary endpoints of adjuvant therapy completion rate and survival.

## Limitations

Several limitations warrant consideration. First, the retrospective design, while mitigated by temporal validation and propensity score matching, cannot eliminate residual confounding. Unmeasured variables—including detailed functional status, socioeconomic factors, and social support networks—may influence both risk stratification and outcomes. Additionally, pre-illness weight was self-reported and subject to recall bias; although we attempted to mitigate this through cross-referencing with medical records and dietitian interviews, some degree of misclassification is inevitable. Second, all patients were of East Asian descent treated at two Beijing centers. Validation in ethnically and geographically diverse cohorts, particularly Western populations with different body composition profiles and practice patterns, is required before widespread adoption. Third, we did not record specific adjuvant chemotherapy regimens (e.g., mFOLFIRINOX vs. gemcitabine-based), which differ substantially in tolerability and may modify the observed mediation pathway. Fourth, quality of life and functional recovery metrics were not assessed, limiting understanding of patient-centered outcomes. Fifth, although we employed state-of-the-art mediation analysis, the observational design means the identified pathway, though biologically plausible and statistically robust, remains hypothesis-generating and would benefit from prospective interventional validation. We provide the complete risk calculation formula in the supplement to facilitate external validation and local calibration.

## Conclusion

We present and validate an early postoperative risk score, tumor type, total muscle index, and weight loss >5%, that identifies periampullary cancer patients with a 22-fold risk of adjuvant therapy failure after pancreaticoduodenectomy. Mediation analysis suggests that 20.9% of the survival disadvantage in high-risk patients may be mediated through major complications leading to adjuvant therapy delay—a modifiable cascade. Available immediately after surgery at no additional cost, this score enables targeted care pathways to improve delivery of multimodal therapy. Prospective trials are needed to confirm its impact on adjuvant completion and survival.

## Data Availability

The raw data supporting the conclusions of this article will be made available by the authors, without undue reservation.
