# Supplementary material for: Muscle index, weight loss, and tumor type predict adjuvant therapy failure in periampullary cancer
Source: Front Nutr. 2026 Jul 27;13:1868235. doi: 10.3389/fnut.2026.1868235 (PMC13453677; doi:10.3389/fnut.2026.1868235)
Supplement: Supplementary file 1 [file Supplementary_File_1.doc]

**Supplementary Material for “Muscle index, weight loss, and tumor type predict adjuvant therapy failure in periampullary cancer”**

This document contains supplementary information to the main manuscript, providing extended methodological details, additional results, and supporting tables and figures. Contents are referenced throughout the main text as follows:

**Supplementary Methods**

*Inclusion and exclusion criteria*

Patients were included if they met the following criteria: (1) histologically confirmed pancreatic head or periampullary carcinoma; (2) no preoperative distant metastases; (3) no arterial tumor attachment; (4) standard PD procedure; (5) age ≥ 18 years; and (6) minimum 6-month follow-up data. Exclusion criteria encompassed: (1) total pancreatectomy; (2) combined multi-organ resection (e.g., partial gastrectomy, colectomy, or nephrectomy); (3) receipt of neoadjuvant chemotherapy or radiotherapy prior to surgery; (4) pathologically proven benign tumors; (5) incomplete clinical data for analysis; and (6) R2 resection (macroscopic residual tumor).

*Nutritional Assessment*

Nutritional status was evaluated using multiple established tools, including the Malnutrition Universal Screening Tool (MUST), Malnutrition Screening Tool (MST), Nutritional Risk Index (NRI), NRS2002, PNI, Controlling Nutritional Status Score (CONUT), Patient-Generated Subjective Global Assessment (PGSGA), and Mini Nutritional Assessment Short Form (MNASF). Our integrated model was also developed and validated in this study. Nutritional assessments were performed by trained dietitians within 7 days preoperatively. All nutritional scores were calculated according to their original published algorithms.

*Assessment of cross-sectional areas and standardization*

Body composition measurements were obtained from routine preoperative contrast-enhanced abdominal computed tomography (CT) scans performed for standard oncological staging. All scans were acquired in the portal venous phase (approximately 60-70 seconds post-contrast injection) using standardized institutional protocols. The cross-sectional areas (cm^2^) of subcutaneous fat (SFA), visceral fat (VFA), and skeletal muscles containing abdominal muscles and psoas and paravertebral muscles, were assessed at the L3 level on serial CT images using commercially available software workstation systems (Advantage Workstation 4.6, GE Healthcare, USA). These assessments were conducted before the surgery by two radiologists with > 5 years of experience independently, who were blinded to the clinic outcomes. Tissue identification was performed using established Hounsfield Unit (HU) thresholds: adipose tissue (both subcutaneous and visceral) was defined as tissue with attenuation between -190 and -30 HU, while skeletal muscle was defined as tissue with attenuation between -29 and +150 HU. Total muscle index (TMI) were then calculated by dividing the skeletal muscle areas by the square of the patient’s height in meters (mm^2^/m^2^).

To ensure measurement consistency, inter-observer reliability was assessed using intraclass correlation coefficients (ICC) for continuous measurements and Cohen's kappa for categorical classifications. ICC values ranged from 0.88 to 0.94, and kappa from 0.82 to 0.89, indicating good to excellent reliability.

*Variable Selection and Assessment*

The initial candidate variable set encompassed demographic, clinical, laboratory, tumor-related, and body composition metrics, including: age, sex, body mass index (BMI), percent weight loss, dietary intake, physical activity, American Society of Anesthesiologists grade (ASA), counts of lymphocyte, total bilirubin levels, albumin levels, prealbumin levels, hemoglobin levels, tumor type, TNM stage, differentiation, R0 resection, serum carbohydrate antigen 19-9 (CA19-9) levels, operative time, intraoperative blood loss, VFA-to-TFA Ratio (VTR), TMI, Neutrophil-to-lymphocyte ratio (NLR), platelet-to-lymphocyte ratio (PLR), lymphocyte-to-monocyte ratio (LMR), and adjuvant chemotherapy delay.

*Follow-up*

Patients were followed according to a standardized institutional protocol, with clinical assessments, laboratory tests and cross-sectional imaging performed at 3-month intervals during the first postoperative year and every 6 months thereafter until 5 years, and annually thereafter. Surveillance included physical examination, serum CA19-9, and contrast-enhanced CT/MRI. The study closing date was January 2025, and endpoints were defined as tumor recurrence, death, or the last documented follow-up.

*Dataset Partition Strategy*

All cases from both centers (N=533) were pooled and chronologically sorted by the surgery date, irrespective of the center. The most recent 30% of this pooled cohort (n=160) formed the temporal validation set. The remaining development set was then randomly split into training (70%, n=261) and internal validation (30%, n=112) sets.

*Statistical Analysis*

Baseline characteristics were summarized as mean ± standard deviation or median (interquartile range) for continuous variables, and frequencies (percentages) for categorical variables, based on distribution normality. Comparisons across training, internal validation, and temporal validation sets were performed using ANOVA or Kruskal-Wallis tests for continuous variables, and chi-square or Fisher's exact tests for categorical variables, as appropriate. Complete-case analysis was used for all regression models, as the 533 included patients had complete data for all variables in the final model (TMI, percent weight loss, tumor type, and survival outcomes). The proportional hazards assumption for the final Cox model was assessed using Schoenfeld residuals; the global test was not significant (χ² = 4.38, df = 3, P = 0.22), confirming that the assumption was not violated. A total of 22 candidate variables were entered into the LASSO-Cox regression model. All analyses were conducted using R version 4.3.0.

*Sample Size Consideration*

Based on the events-per-variable (EPV) principle requiring 10 events per predictor, our training set with 140 events provided sufficient power for model development with up to 14 candidate predictors. With 22 initial candidate variables, we employed LASSO regression to reduce dimensionality while maintaining statistical robustness. Given that the final model contained only 3 predictors, the effective EPV ratio exceeded 40, ensuring high stability and minimal overfitting risk. Although the training set contained 140 events (slightly below the recommended 150-200 for novel score development), the total cohort had 261 events across all datasets, and the model demonstrated consistent performance in temporal validation, supporting its robustness.

*Variable Selection and Model Development*

The variables were screened using LASSO-Cox regression with 10-fold cross-validation in the training set (n=261). The optimal penalty parameter (λ) was selected using the one-standard-error rule (λ.1se=0.182) to balance model complexity and predictive accuracy (eFigure 1). Selected variables were standardized for clinical interpretation: TMI was analyzed per 100-unit increase, and percent weight loss was dichotomized at the clinically relevant 5% threshold.

*Model Performance Assessment*

Discrimination was quantified using Harrell's C-index with 95% confidence intervals (CIs) derived from 1000 bootstrap resamples. Calibration was assessed visually using calibration curves with smoothed loess fits and statistically using the Hosmer-Lemeshow test. Clinical utility was evaluated through decision curve analysis across a range of threshold probabilities (0-50%).

*Validation Strategy*

A dual validation approach was employed: (1) internal validation using a random 70:30 split of the development set, and (2) external temporal validation using the most recent 30% of patients (n=160) to assess model generalizability to future patients.

*Incremental Value Assessment*

The added value of the nutritional-body composition model was tested against: (1) a baseline clinical model containing tumor type only, and (2) established nutritional scores. Improvement was quantified using continuous net reclassification improvement (NRI) and integrated discrimination improvement (IDI) with 95% CIs.

*Risk Stratification and Propensity Score Matching*

In the temporal validation set, patients were stratified into low-, intermediate-, and high-risk groups based on tertiles of nomogram-predicted scores. To isolate the effect of the integrative nutritional risk from other strong prognostic factors and to create balanced cohorts for comparing subsequent postoperative outcomes (complications, adjuvant delays), propensity score matching (1:1 ratio, caliper=0.2 SD) was performed using logistic regression with age, sex, TNM stage, tumor differentiation, vascular invasion, and R0 resection as matching variables. Balance was assessed using standardized mean differences (<0.1 considered adequate).

*Mediation Analysis*

To explore the sequential pathway linking high nutritional risk to poor DFS, we performed a formal sequential mediation analysis. We hypothesized a pathway where high nutritional risk is associated with postoperative complications, which in turn are associated with adjuvant therapy delays, ultimately associated with recurrence.

This analysis was conducted on the full temporal validation cohort (n=160) to preserve statistical power and sample representativeness. We employed a counterfactual-based framework using the mediation package in R. Three regression models were specified:

Mediator Model (Complications): Logistic regression with postoperative complications (binary) as the outcome, with high nutritional risk (binary, based on model tertiles) as the exposure, and adjusted for age, sex, TNM stage, tumor differentiation, and operative duration.

Mediator Model (Adjuvant Delay): Logistic regression with adjuvant therapy delay (binary) as the outcome, with postoperative complications as the exposure and high nutritional risk as a covariate, adjusted for the same set of clinical variables as in model 1.

Outcome Model (DFS): Cox proportional hazards model with DFS as the outcome, including high nutritional risk, postoperative complications, and adjuvant therapy delay as predictors, again adjusted for age, sex, TNM stage, and tumor differentiation.

The total, direct (path not through the specified mediators), and indirect effects were estimated. The proportion of the total effect mediated through the sequential pathway was calculated with 95% CIs derived from 1000 bootstrap simulations. We emphasize that this analysis estimates mediation effects under the strong assumptions of no unmeasured confounding of (a) the mediator-outcome relationships, (b) the exposure-mediator relationships, and (c) the exposure-outcome relationship. Therefore, while providing strong statistical evidence for a plausible and clinically actionable biological pathway, these results should be interpreted as supporting, rather than definitively proving, causality.

**Supplementary Results**

This section provides extended results corresponding to the analyses reported in the main text.

*Patient Cohort and Characteristics*

Comparative analysis revealed well-balanced characteristics between training and internal validation sets for most variables, while some expected temporal variations were observed in the temporal validation set, particularly in ASA classification (P<0.001) and TNM staging distribution (P=0.006), reflecting evolution in clinical practice and patient selection over time.

Major postoperative complications occurred with remarkable consistency across all three datasets: 29.1% (76/261), 29.5% (33/112), and 27.5% (44/160) in training, internal validation, and temporal validation sets, respectively (P=0.928). Adjuvant chemotherapy was not received or was delayed in 34.9% (91/261), 47.3% (53/112), and 36.9% (59/160) of cases (P=0.073). Notably, while complication rates showed no temporal variation, adjuvant therapy omission/delay rates displayed a distinct pattern: the internal validation cohort exhibited the highest rate (47.3%), whereas training and temporal validation cohorts demonstrated more comparable rates (34.9% vs 36.9%). This finding suggests that factors beyond surgical complications may influence adjuvant therapy delivery, setting the stage for our subsequent mediation analysis examining the interplay between nutritional risk, complications, and treatment delays.

*Prediction Model Development and Nomogram Construction*

The risk score generated by the model exhibited a perfect gradient aligned with known tumor biology (P for trend <0.001). Patients with PDAC exhibited the highest median risk scores (mean = 1.83, IQR 1.24-2.45), followed by CDP (mean = 1.02, IQR 0.42-1.72) and DBC (mean = 1.01, IQR 0.41-1.65), while AC patients showed the lowest risk (mean = 0.16, IQR -0.32-0.62) (P<0.001). This alignment provides direct biological validation that our model successfully contextualizes host physiology within the specific tumor type. Although the individual HRs for DBC and CDP did not reach statistical significance, the integrated model's continuous risk score perfectly aligned with the established tumor aggressiveness gradient (PDAC > DBC/CDP > AC). This demonstrates the model's core strength: it contextualizes host factors (TMI, weight loss) within tumor biology, generating a composite metric that more accurately reflects prognosis than any variable in isolation.

To explore model applicability across histologies, we assessed discrimination within each major tumour subtype in the temporal validation set. The model maintained promising discriminatory ability across all subtypes, with C-indices of 0.828 (CDP), 0.774 (DBC), 0.728 (PDAC), and 0.700 (AC). Notably, the wide confidence intervals for CDP and AC, attributable to smaller sample sizes, warrant cautious interpretation and highlight the need for validation in larger subtype-specific cohorts.

*Internal and Temporal Validation of Model Performance*

Time-dependent receiver operating characteristic (ROC) analysis in the temporal validation set revealed robust short-to-medium term predictive accuracy, with area under the curve (AUC) values of 0.739, 0.791, and 0.764 for 1-, 2-, and 3-year DFS prediction, respectively (eFigure 2). Both calibration curves and decision curve analysis demonstrated favorable model performance, with good agreement between predicted and observed probabilities and superior net benefit compared to default strategies across clinically relevant threshold probabilities (eFigures 3-4), supporting the model's clinical utility for periampullary carcinoma patients.

*Incremental Value and Comparative Performance Analysis*

The model outperformed the best-performing conventional nutritional score, MNASF (C-index: 0.612, 95% CI: 0.530-0.693), by 14.7% relative improvement, while showing even greater advantages over other established tools including PGSGA (27.2% relative improvement), NRS2002 (30.1% relative improvement), and PNI (30.5% improvement). Notably, the model maintained consistent superiority across the entire spectrum of nutritional assessment methodologies, with all comparative differences achieving statistical significance at P < 0.001.

The hierarchical performance ranking established our model as the superior discriminative tool, followed by MNASF, while traditional inflammatory ratios such as NLR demonstrated the lowest predictive accuracy (C-index: 0.464, 95% CI: 0.383-0.545). This systematic evaluation underscores the critical importance of integrating preoperative nutritional and body composition metrics with postoperative pathological findings, providing enhanced prognostic precision for surgical patients.

*Risk Stratification and Association with Postoperative Outcomes*

*1) Robust Risk Stratification and Survival Disparities*

To evaluate the clinical applicability of our risk stratification in routine practice, we examined its prognostic performance within each tumor subtype in the temporal validation cohort. Despite inherent differences in baseline prognosis, the risk score effectively stratified patients within each histologic category. In PDAC patients (n=68), high-risk designation (based on the overall cohort's upper tertile cutoff) identified a subgroup with particularly poor outcomes (median DFS: 14.2 vs. 25.6 months, HR=2.87, 95% CI: 1.52-5.42, P=0.001). Similarly, in DBC patients (n=52), high-risk patients showed significantly worse DFS compared to low-risk counterparts (HR=2.15, 95% CI: 1.08-4.28, P=0.029). These findings demonstrate that our integrative risk assessment provides actionable prognostic information even within homogeneous tumor categories. The clear separation of risk scores across stratification groups further validated the model's discriminative ability (eFigure 8).

2) *Propensity-Score Matched Validation*

As intended by our study design, the fundamental difference between the matched groups remained the high vs. low nutritional risk status, as evidenced by persistent significant differences in TMI and percent weight loss (both P<0.001; eTable 1), confirming that the matching process selectively balanced clinical confounders while preserving the nutritional risk gradient central to our hypothesis. The concomitant imbalance in tumor type distribution is not a flaw but an expected validation, as it reflects the intrinsic integration of tumor biology into our risk construct.

*Mediation Analysis: Elucidating the Complication-to-Treatment Delay Pathway*

Building on the observation that adjuvant therapy delays (34.9-47.3%) substantially exceeded complication rates (27.5-29.5%) across all cohorts, we hypothesized that complications might serve as a critical mediator disrupting treatment continuity. Formal sequential mediation analysis provided compelling statistical evidence for the proposed mechanistic pathway linking peri-operative nutritional risk to impaired survival outcomes through postoperative complications and subsequent adjuvant therapy delays.

The total association of high nutritional risk with reduced DFS was substantial (HR = 5.88, 95% CI: 2.56-13.48, P < 0.001). After accounting for the mediation pathway through complications and adjuvant delays, the direct effect remained significant but attenuated (HR = 4.06, 95% CI: 1.71-9.67, P = 0.015). In all, these findings establish a clear mechanistic pathway: preoperative nutritional compromise → increased postoperative morbidity → delayed adjuvant therapy → impaired survival. Crucially, nearly one-fifth (20.9%) of the total detrimental effect of nutritional risk on DFS was mediated through this sequential pathway. This elevates preoperative nutritional assessment from a simple prognostic indicator to a key component in understanding—and potentially interrupting—the clinical cascade that leads to poor cancer outcomes.

**eFigures and eTables**

**eFigure 1. Variable selection using LASSO-Cox regression**

LASSO-Cox regression with 10-fold cross-validation was used to select the most predictive variables for disease-free survival. The vertical dashed line indicates the optimal lambda value (lambda.1se) that minimizes the cross-validation error while maintaining model parsimony. Variables with non-zero coefficients at this lambda value were selected for the final model.

**eFigure 2. Time-dependent receiver operating characteristic curves**

Area under the curve (AUC) values demonstrate model discrimination performance for 1-year (AUC = 0.739), 2-year (AUC = 0.791), and 3-year (AUC = 0.764) disease-free survival prediction in the temporal validation cohort. The dashed diagonal line represents random chance performance (AUC = 0.5). ROC, receiver operating characteristic; TMI, total muscle index; AUC, area under the curve

**eFigure 3. Calibration curves for survival probability prediction**

The plots compare predicted probabilities (x-axis) with observed outcomes (y-axis) for 1-, 2-, and 3-year disease-free survival. Perfect calibration would follow the 45-degree diagonal line. The close adherence of calibration curves to the diagonal indicates excellent agreement between predicted probabilities and actual observed survival rates across all timepoints. DFS, disease-free survival

**eFigure 4. Decision curve analysis for clinical utility assessment**

The net benefit of using our model for 1-, 2-, and 3-year disease-free survival is compared against "treat all" (gray line) and "treat none" (horizontal line at zero) strategies across a range of threshold probabilities. The model demonstrates superior clinical utility when our model is above both comparison strategies, indicating it provides better clinical decision support across most clinically relevant risk thresholds. DFS, disease-free survival

**eFigure 5. Incremental prognostic value of the tumor-host integrative model**

Comparison of discrimination performance between the baseline clinical model (incorporating tumor type) and our comprehensive model. The integrative model showed a statistically significant improvement in C-index (ΔC-index = 0.105, P < 0.001). Error bars indicate 95% confidence intervals.

**eFigure 6. Statistical metrics of the integrative model's incremental prognostic value over the baseline model.**

Bar plots quantify the improvement in predictive performance when adding total muscle index and percent weight loss to a baseline model containing only tumor type. All metrics demonstrate statistically significant enhancement: Integrated Discrimination Improvement (IDI) = 0.150 (P = 0.001), ΔC-index = 0.106 (P = 0.012), and Net Reclassification Improvement (NRI) = 0.826 (P = 0.002). The likelihood ratio test (LRT) confirmed a superior overall model fit (χ^2^ = 41.01, P < 0.001).

**eFigure 7. Comparison of discrimination performance among different nutritional assessment tools**

The bar plot displays the C-index with 95% confidence intervals for our model and established nutritional assessment tools in the temporal validation cohort (n=160). Error bars represent 95% confidence intervals, and all comparisons against our model were statistically significant (P < 0.001). MUST, malnutritional universal screening tool; MST, malnutrition screening tool; NRI, nutritional risk index; NRS 2002, nutritional risk screening score 2002; PGSGA, patient generated subjective global assessment; PNI, prognostic nutritional index; CONUT, controlling nutritional status score; MNASF, mini nutritional assessment short form; NLR, neutrophil-to-lymphocyte count ratio; PLR, platelet-to-lymphocyte count ratio; LMR, lymphocyte-to-monocyte count ratio

**eFigure 8. Distribution of model-predicted risk scores across stratification groups in the temporal validation cohort.**

Violin plots depict the probability density of risk scores for patients stratified into low-, intermediate-, and high-risk groups based on score tertiles. Boxplots within each violin show the median (central line) and interquartile range (box boundaries). Individual data points are overlaid. Risk scores differed significantly across groups (Kruskal-Wallis P < 0.001)

eTable 1. Clinical and Pathological Characteristics of Patients Across Cohorts

| Variable | Level | Training | Internal Validation | Temporal Validation | P Value |
| --- | --- | --- | --- | --- | --- |
| Age |  | 64 ± 9.74 | 63 ± 10.09 | 64.66 ± 10.08 | 0.201 |
| Sex |  |  |  |  | 0.772 |
|  | Male | 152 (58.2%) | 67 (59.8%) | 89 (55.6%) |  |
|  | Female | 109 (41.8%) | 45 (40.2%) | 71 (44.4%) |  |
| BMI |  | 23.5 ± 2.9 | 23.42 ± 3.34 | 22.82 ± 2.84 | 0.113 |
| Tumor type |  |  |  |  | 0.361 |
|  | AC | 48 (18.4%) | 11 (9.8%) | 28 (17.5%) |  |
|  | DBC | 96 (36.8%) | 42 (37.5%) | 52 (32.5%) |  |
|  | PDAC | 93 (35.6%) | 49 (43.8%) | 68 (42.5%) |  |
|  | CDP | 24 (9.2%) | 10 (8.9%) | 12 (7.5%) |  |
| TNM stage |  |  |  |  | 0.006 |
|  | III | 74 (28.4%) | 26 (23.2%) | 20 (12.5%) |  |
|  | II | 133 (51%) | 57 (50.9%) | 99 (61.9%) |  |
|  | I | 53 (20.3%) | 28 (25%) | 40 (25%) |  |
|  | 0 | 1 (0.4%) | 1 (0.9%) | 1 (0.6%) |  |
| Tumor size (cm) |  | 2.76 ± 1.41 | 2.72 ± 1.19 | 2.74 ± 1.26 | 0.904 |
| Differentiation |  |  |  |  | 0.188 |
|  | Well | 49 (18.8%) | 20 (17.9%) | 32 (20%) |  |
|  | Moderately | 157 (60.2%) | 61 (54.5%) | 79 (49.4%) |  |
|  | Poorly | 55 (21.1%) | 31 (27.7%) | 49 (30.6%) |  |
| Venous invasion |  |  |  |  | 0.507 |
|  | No | 209 (80.1%) | 84 (75%) | 128 (80%) |  |
|  | Yes | 52 (19.9%) | 28 (25%) | 32 (20%) |  |
| R0 resection |  |  |  |  | 0.015 |
|  | Yes | 240 (92%) | 108 (96.4%) | 157 (98.1%) |  |
|  | No | 21 (8%) | 4 (3.6%) | 3 (1.9%) |  |
| CA199 (ng/mL) |  | 834.04 ± 2267.69 | 799.35 ± 2225.62 | 522.45 ± 1619.36 | 0.264 |
| TB (g/L) |  | 109.93 ± 106.5 | 109.92 ± 99.94 | 108.11 ± 96.95 | 0.791 |
| ALB (g/L) |  | 35.27 ± 5.58 | 34.82 ± 5.58 | 36.06 ± 4.95 | 0.195 |
| PAB (g/L) |  | 0.17 ± 0.08 | 0.16 ± 0.07 | 0.16 ± 0.07 | 0.472 |
| HGB (g/L) |  | 118.8 ± 18.8 | 116.7 ± 18 | 117.03 ± 18.74 | 0.713 |
| NLR |  | 3.45 ± 3.36 | 3.5 ± 2.31 | 3.61 ± 3.78 | 0.544 |
| PLR |  | 187.11 ± 96.28 | 199.42 ± 105 | 198.47 ± 118.94 | 0.553 |
| LMR |  | 4.13 ± 2.35 | 3.81 ± 1.84 | 3.63 ± 1.67 | 0.259 |
| LY (10^9^/L) |  | 1.53 ± 0.69 | 1.42 ± 0.57 | 1.45 ± 0.58 | 0.362 |
| VFA (cm^2^) |  | 9079.51 ± 4496.58 | 9118.61 ± 4995.6 | 8795.1 ± 5305.53 | 0.519 |
| SFA (cm^2^) |  | 9344.62 ± 4645.04 | 9035.35 ± 4822.26 | 8833.08 ± 4814.79 | 0.326 |
| TMI (mm^2^/m^2^) |  | 3917.68 ± 979.35 | 3750.13 ± 956.51 | 3862.99 ± 953.38 | 0.325 |
| VTR |  | 0.49 ± 0.12 | 0.5 ± 0.13 | 0.48 ± 0.15 | 0.711 |
| PWL |  | 0.05 ± 0.04 | 0.05 ± 0.04 | 0.05 ± 0.03 | 0.808 |
| Weight loss (kg) |  | 3.28 ± 2.75 | 3.47 ± 2.64 | 3.32 ± 2.39 | 0.742 |
| Diet |  |  |  |  | 0.129 |
|  | Good | 230 (88.1%) | 96 (85.7%) | 137 (85.6%) |  |
|  | Moderate | 20 (7.7%) | 13 (11.6%) | 10 (6.2%) |  |
|  | Poor | 11 (4.2%) | 3 (2.7%) | 13 (8.1%) |  |
| Physical activity |  |  |  |  | 0.191 |
|  | Good | 239 (91.6%) | 104 (92.9%) | 141 (88.1%) |  |
|  | Moderate | 15 (5.7%) | 7 (6.2%) | 9 (5.6%) |  |
|  | Poor | 7 (2.7%) | 1 (0.9%) | 10 (6.2%) |  |
| ASA score |  |  |  |  | <0.001 |
|  | 1 | 21 (8%) | 7 (6.2%) | 5 (3.1%) |  |
|  | 2 | 101 (38.7%) | 43 (38.4%) | 99 (61.9%) |  |
|  | 3 | 88 (33.7%) | 41 (36.6%) | 46 (28.7%) |  |
|  | 4 | 51 (19.5%) | 21 (18.8%) | 10 (6.2%) |  |
| OT (h) |  | 9.93 ± 2.28 | 9.79 ± 2.24 | 9.43 ± 2.48 | 0.088 |
| IBL (ml) |  | 586.78 ± 304.56 | 562.5 ± 402.95 | 558.12 ± 365.62 | 0.069 |

BMI, body mass index; AC , ampullary carcinoma; DBC , distal bile duct carcinoma; PDAC , pancreatic ductal adenocarcinoma; CDP, carcinoma of the duodenal papilla; TB, total bilirubin; ALB, albumin; PAB, prealbumin; HGB, hemoglobin; NLR, neutrophil-to-lymphocyte ratio; PLR, platelet-to-lymphocyte ratio; LMR, lymphocyte-to-monocyte ratio; LY, lymphocyte; VFA, visceral fat area; SFA, subcutaneous fat area; VTR, VFA to total fat area; TMI, total muscle index; ASA, American Society of Anesthesiologists; OT, Operative time ; IBL, intraoperative blood loss

Event rates for disease-free survival were 140/261 (53.6%) in the training set, 66/112 (58.9%) in the internal validation set, and 55/160 (34.4%) in the temporal validation set. The chronological split explains the differing event rates, with the most recent patients forming the temporal validation set.

eTable 2. Complete List of Candidate Variables Evaluated in LASSO-Cox Regression

| Category | Variable | LASSO_Coefficient | Selected |
| --- | --- | --- | --- |
| Body Composition | Total Muscle Index (mm²/m²) | -0.0004 | Yes |
| Tumor-related | Tumor Type: Pancreatic Ductal Adenocarcinoma | 0.4874 | Yes |
| Nutritional | Weight Loss (>5% vs ≤5%) | 0.0887 | Yes |
| Demographic | Age (years) | 0.0000 | No |
| Nutritional | Albumin (g/L) | 0.0000 | No |
| Surgical/Perioperative | ASA Grade 2 | 0.0000 | No |
| Surgical/Perioperative | ASA Grade 3 | 0.0000 | No |
| Surgical/Perioperative | ASA Grade 4 | 0.0000 | No |
| Other | Body Mass Index (kg/m²) | 0.0000 | No |
| Tumor-related | CA19-9 (U/mL) | 0.0000 | No |
| Nutritional | Dietary Intake (Level 2) | 0.0000 | No |
| Nutritional | Dietary Intake (Level 3) | 0.0000 | No |
| Inflammatory/Hematological | Hemoglobin (g/L) | 0.0000 | No |
| Surgical/Perioperative | Intraoperative Blood Loss (mL) | 0.0000 | No |
| Inflammatory/Hematological | Lymphocyte-to-Monocyte Ratio | 0.0000 | No |
| Inflammatory/Hematological | Lymphocyte Count (×10⁹/L) | 0.0000 | No |
| Inflammatory/Hematological | Neutrophil-to-Lymphocyte Ratio | 0.0000 | No |
| Surgical/Perioperative | Operative Time (hours) | 0.0000 | No |
| Nutritional | Physical Activity (Level 2) | 0.0000 | No |
| Nutritional | Physical Activity (Level 3) | 0.0000 | No |
| Inflammatory/Hematological | Platelet-to-Lymphocyte Ratio | 0.0000 | No |
| Nutritional | Prealbumin (g/L) | 0.0000 | No |
| Tumor-related | R0 Resection (Yes vs No) | 0.0000 | No |
| Demographic | Sex (Male vs Female) | 0.0000 | No |
| Tumor-related | Total Bilirubin (μmol/L) | 0.0000 | No |
| Tumor-related | Tumor Type: Carcinoma of Duodenal Papilla | 0.0000 | No |
| Tumor-related | Tumor Type: Distal Bile Duct Carcinoma | 0.0000 | No |
| Body Composition | VFA-to-TFA Ratio | 0.0000 | No |

ASA, American Society of Anesthesiologists; VFA, visceral fat area; TFA, total fat area; TMI, total muscle index. LASSO coefficients are reported on the original scale of each variable as entered into the model. For categorical variables, the first level (e.g., ASA Grade 1, Dietary Intake Level 1, Physical Activity Level 1, Ampullary Carcinoma) serves as the reference category and does not appear in the table. TMI was standardized per 100 units increase (TMI/100) for clinical interpretability. Variables with non-zero coefficients at the selected lambda value were retained in the final model (marked "Yes").

eTable 3: Model Discrimination Performance (C-index) Across Datasets

| Dataset | Patients | Events | C-index | 95% CI |
| --- | --- | --- | --- | --- |
| Training Set | 261 | 139 | 0.779 | 0.745 - 0.813 |
| Internal Validation Set | 112 | 67 | 0.724 | 0.67 - 0.779 |
| Temporal Validation Set | 160 | 55 | 0.702 | 0.618 - 0.786 |

The overall 1-year mortality rate in the total cohort (N=533) was 55.7% (95% CI: 51.5%-59.9%). Events represent recurrence or death for disease-free survival analysis. CI, confidence interval

eTable 4. Baseline Characteristics After Propensity Score Matching (49 pairs, n=98)

|  |  | Risk Group | |  |  |
| --- | --- | --- | --- | --- | --- |
| Variable | Level | Non-High Risk | High Risk | Standardized Difference | P Value |
| Age (years) |  | 65.1 ± 10.4 | 65.1 ± 9.7 | 0.008 | 0.9681 |
| Sex | Male | 26 (53.1%) | 28 (57.1%) | 0.082 | 0.8391 |
|  | Female | 23 (46.9%) | 21 (42.9%) | 0.082 |  |
| Body Mass Index (kg/m²) |  | 22.6 ± 2.7 | 23.4 ± 2.7 | 0.311 | 0.1271 |
| Tumor Type | AC | 15 (30.6%) | 1 (2.0%) | 0.838 | 0.0002 |
|  | CDP | 6 (12.2%) | 5 (10.2%) | 0.065 |  |
|  | DBC | 12 (24.5%) | 11 (22.4%) | 0.048 |  |
|  | PDAC | 16 (32.7%) | 32 (65.3%) | 0.691 |  |
| TNM Stage | I | 16 (32.7%) | 13 (26.5%) | 0.134 | 0.6225 |
|  | II | 27 (55.1%) | 32 (65.3%) | 0.21 |  |
|  | III | 6 (12.2%) | 4 (8.2%) | 0.135 |  |
| Differentiation | Well | 10 (20.4%) | 10 (20.4%) | 0 | 1 |
|  | Moderately | 26 (53.1%) | 26 (53.1%) | 0 |  |
|  | Poorly | 13 (26.5%) | 13 (26.5%) | 0 |  |
| Albumin (g/L) |  | 35.7 ± 5.2 | 37.1 ± 4.3 | 0.3 | 0.1403 |
| Total Muscle Index |  | 4345.5 ± 803.2 | 3030.2± 511.9 | 1.953 | 0 |
| Percent Weight Loss (%) |  | 3.9 ± 3.2 | 6.5 ± 3.3 | 0.808 | 0.0001 |

AC, ampullary carcinoma; DBC, distal bile duct carcinoma; PDAC, pancreatic ductal adenocarcinoma; CDP, carcinoma of the duodenal papilla

Note: Tumor type, total muscle index, and percent weight loss were intentionally excluded from the propensity score matching variables, as they are integral components of the exposure (integrative nutritional risk). Their persistent imbalance after matching validates that the ‘high-risk’ phenotype represents a synergistic combination of tumor biology and host physiology.
